# SUV39H1 is a novel biomarker targeting oxidative phosphorylation in hepatitis B virus-associated hepatocellular carcinoma

**DOI:** 10.1186/s12885-023-11633-4

**Published:** 2023-11-28

**Authors:** Yanping Zhang, Wanwen Lao, Kaming Yang, Xinyi Kong, Yuetong Li, Xin Yu, Xumeng Wang, Yang Liu, Zhenlin Li, Yilin Deng, Shuping Nie, Changlong Bi, Chao Wu, Aixia Zhai

**Affiliations:** 1 Department of Laboratory Medicine, The Eighth Affiliated Hospital, Sun Yat-sen University, Shenzhen, 518033 China; 2 Department of Endocrinology, The Eighth Affiliated Hospital, Sun Yat-sen University, Shenzhen, 518033 China; 3 Department of Microbiology, Harbin Medical University, Harbin, 150081 China

**Keywords:** SUV39H1, Hepatocellular carcinoma, Hepatitis B virus, Oxidative phosphorylation, Biomarker

## Abstract

**Background:**

As a histone methyltransferase, suppressor of variegation 3–9 homolog 1 (SUV39H1) plays an important role in the occurrence and development of cancer. To explore the mechanism and biological function of SUV39H1 in hepatitis B virus-associated hepatocellular carcinoma (HBV-HCC) can gain an insight into the pathogenesis of HBV-HCC.

**Methods:**

The effect of HBV infection on SUV39H1 in hepatoma cells was detected. CCK-8, colony growth assay and wound healing assay were used to assess the proliferation and migration of HBV-positive hepatoma cells. RNA sequencing (RNA-seq) was applied to find differential genes and enriched pathways. The serum SUV39H1 level in HBV-HCC patients was detected and its correlation with clinical indicators was analyzed.

**Results:**

SUV39H1 was increased by HBV infection and promoted the proliferation and migration of hepatoma cells. SUV39H1 could upregulate the expression of mitochondrial oxidative phosphorylation (OXPHOS) pathway-related genes. OXPHOS pathway inhibitors could reduce the capacity of proliferation and migration of hepatoma cells after overexpressing SUV39H1. Serum SUV39H1 levels were higher in chronic hepatitis B (CHB) patients than in healthy controls and higher in HBV-HCC patients than in CHB patients. In the diagnosis of HCC, the predictive value of SUV39H1 combined with alpha-fetoprotein (AFP) was better than that of AFP alone.

**Conclusion:**

SUV39H1 is regulated by HBV infection and promotes the proliferation and migration of hepatoma cells by targeting OXPHOS pathway. It indicates that SUV39H1 may be a new biomarker of the diagnosis of HCC.

**Supplementary Information:**

The online version contains supplementary material available at 10.1186/s12885-023-11633-4.

## Introduction

Primary liver cancer is one of the most common cancers worldwide and the third leading cause of cancer-related death [1]. Hepatocellular carcinoma (HCC) accounts for about 90% of the total cases of primary liver cancer [2]. Known major risk factors for HCC include hepatitis B virus (HBV) infection, hepatitis C virus (HCV) infection, excessive alcohol consumption, aflatoxin, obesity, diabetes mellitus, and non-alcoholic fatty liver disease, among which HBV infection plays an essential role [3, 4]. Therefore, it is particularly imperative to explore the pathogenesis of HBV-associated HCC (HBV-HCC).

Histone methylation is one of the most complex histones post-translational modifications. It can be methylated in lysine and arginine residues, playing an important role in the progression of HCC [5]. Suppressor of variegation 3–9 homolog 1 (SUV39H1) is a histone lysine methyltransferase responsible for the trimethylation of histone 3 lysine 9 (H3K9) and regulates heterochromatin formation to inhibit transcription [6, 7]. It has been reported that the role of SUV39H1 in cancer is a double-edged sword. SUV39H1 acts as a tumor suppressor in cervical cancer, parathyroid cancer and leukemia, but plays a cancer-promoting role in colorectal cancer and melanoma [8–12]. Recently, studies have shown that SUV39H1 is upregulated in HCC patient samples and is associated with HCC recurrence rate [13, 14]. It has been reported that AIFM2 can enhance mitochondrial biogenesis through activation of SIRT1/PGC-1α signaling to promote hepatocellular carcinoma metastasis [15]. Moreover, SIRT1 can interact directly with SUV39H1, and contribute to elevated levels of SUV39H1 activity [16]. However, the role and mechanism of SUV39H1 in the progression of liver cancer remains largely elusive.

Tumor initiation and progression require metabolic reprogramming [17]. However, previous studies mainly focused on the changes in tumor metabolism, such as glycolysis, while oxidative phosphorylation (OXPHOS) was rarely reported. OXPHOS is a fundamental mitochondrial metabolic process that produces adenosine triphosphate (ATP) by transporting electrons to a series of transmembrane protein complexes, called electron transport chains (ETC) in the inner mitochondrial membrane [18]. Recent studies have shown that mitochondrial energy pathways are reprogrammed and tumor cells metabolic signals are upregulated to promote and meet the metabolic needs of cancer growth and proliferation in highly aggressive tumors [19]. Therefore, OXPHOS is also being considered as an emerging target for cancer therapy. We found that SUV39H1 overexpression led to increased transcript levels of most genes in the oxidative phosphorylation pathway in HCC by transcriptome sequencing. In addition, increased intracellular ATP production was detected after SUV39H1 overexpression in HCC cells, implying altered cellular metabolism. Since the relationship between SUV39H1 and oxidative phosphorylation pathway has not been reported, we investigated the effect of SUV39H1 on the oxidative phosphorylation pathway of HCC to provide a new pathogenic mechanism for HCC.

In this study, we examined SUV39H1 expression in HBV infected hepatoma cells and determined the effect of SUV39H1 on proliferation and migration of hepatoma cells. Further, we explored the underlying molecular pathway mediated by SUV39H1 in HCC progression. In addition, we investigated serum SUV39H1 levels in healthy controls, CHB patients and HBV-HCC patients. And we analyzed the correlation of various liver function indicators with SUV39H1. Moreover, we compared the diagnostic potential of AFP and SUV39H1 in patients with HBV-HCC. In conclusion, our work demonstrated the function and molecular mechanism of SUV39H1 and gained an insight into the diagnostic potential of SUV39H1in HBV-HCC patients.

## Materials and methods

### Microarray data

The gene expression profiling dataset GSE121248 was obtained from the gene expression omnibus (GEO, https://www.ncbi.nlm.nih.gov/geo/), which based on Platforms GPL570 (Affymetrix Human Genome U133 Plus 2.0 Array). Totally 70 tumor tissues from chronic hepatitis B (CHB) induced HCC and 37 adjacent normal tissues were obtained.

### Survival analysis

Kaplan-Meier analysis was performed for disease free survival (DFS) in the Gene Expression Profiling Interactive Analysis (GEPIA) database (http://gepia2.cancer-pku.cn/#analysis). And the low SUV39H1 group was based on 20% cutoff-low expression value of SUV39H1 in HCC patients, whereas the high SUV39H1 group based on 40% cutoff-high expression value.

### Plasmids and small interference RNA (siRNA)

The HBV plasmid was a pGEM-4Z vector carrying 1.3 copies of the HBV genome. And the SUV39H1 overexpression plasmid was that SUV39H1 (NM_001282166.1) subcloned into pcDNA3.1 vector. They were obtained from MiaoLing Plasmid Platform (Wuhan, China). SUV39H1 siRNA (si-SUV39H1) and the negative control siRNA (si-NC) were designed and synthesized by Genepharm (Shanghai, China). The siRNA sequences were listed in Table S1.

### Cell culture and transfection

HepG2 cells were derived from human hepatoblastoma and HepG2.215 cells were HepG2 cells that stabilized the whole gene sequence of HBV, which could stably produce infectious HBV particles. They were both purchased from iCell Bioscience Inc (Shanghai, China). Hep3B cells were human hepatocellular carcinoma cells, which contained an integrated hepatitis B virus genome and could synthesize hepatitis B surface antigen (HBsAg) [20]. All cells were cultured in Dulbecco’s modified Eagle’s medium (DMEM) (Gibco, USA) containing 10% fetal bovine serum (FBS) (Gibco, USA) and 1% penicillin-streptomycin (Gibco, USA) and maintained in a 5% CO_2_ atmosphere at 37℃. Plasmids and a final concentration of 100 nM siRNA was transfected into the cells with Lipofectamine 2000 (Invitrogen, USA).

Lentiviruses containing LV-NC (negative control) and LV-oe-SUV39H1 were constructed using pLV[Exp]-EGFP:T2A:Puro-EF1A vector by VectorBuilder Inc (Guangzhou, China). HepG2 cells were exposed to recombinant lentivirus with 8 µg/mL polybrene. The successfully infected cells were selected with 2 µg/mL puromycin. Chaetocin was an inhibitor of the SUV39 family to target H3K9. The cells were treated with Chaetocin (Selleckchem, USA) 50 nM for 24 h. Rotenone and Oligomycin were inhibitors of OXPHOS pathway and cells were treated with Rotenone (MCE, USA) 0.5 µM for 24 h or Oligomycin (MCE, USA) 1 µM for 24 h.

### Quantitative real-time PCR (qPCR)

Total RNA samples were extracted from treated cells using TRIzol (Invitrogen, USA). For each sample, 500 ng of total RNA was reverse transcribed into cDNA with Evo M-MLV RT Premix for qPCR (Accurate Biology, China), and amplified with SYBR Green premix Pro Taq HS qPCR Kit (Accurate Biology, China) using LightCycler 96 instrument (Roche, Switzerland). The PCR primers used were listed in Table S1 [21]. The relative expression was determined using the 2^−ΔΔCT^ method and transcript levels were normalised to the levels of GAPDH mRNA expression.

### Western blot (WB)

Total protein samples were separated on a 10% SDS-PAGE gel and transferred to PVDF membrane (Millipore, USA), the membranes were blocked with 5% skim milk. The blots were cut and then incubated with the primary antibodies of anti-SUV39H1 (Cell Signaling Technology, USA, #8729) and anti-GAPDH (Cell Signaling Technology, USA, #5174) respectively. Horseradish peroxidase (HRP)-conjugated anti-rabbit IgG antibody was used as a secondary antibody, and SuperSignal West Femto Maximum Sensitivity Substrate (Thermo, USA) was used for chemiluminescent detection. All blots including all replicates with clear membrane edges were provided in the Supplementary Information file.

### Cell counting Kit-8 (CCK-8)

Hep3B cells were seeded into 96-well plates with 5,000 cells each well. The CCK-8 reagent (Bioscience, China) was added and incubated for 1–2 h. The OD values were measured at 450 nm using a microplate spectrophotometer (Thermo, USA) at 0 h, 24 h, 48 h, 72 h, 96 h, respectively.

### Colony growth assay

Hep3B cells were seeded into 6-well plates with 1000 or 2500 cells each well and cultured in an incubator with medium changes every 3 days. When visible white granules of cells are observed, the colonies were fixed, stained, photographed and counted.

### Wound healing assay

Hep3B cells were seeded into 6-well plates and transfected with the plasmids and siRNAs of SUV39H1 for 48 h. The cell monolayer was scraped by sterile 10 µL plastic pipette tips, and then cells were washed twice. 1% FBS culture medium was added at different time periods (0 h, 24 h, or 48 h). Images of wound closure were recorded by an inverted microscope and evaluated using ImageJ software (National Institutes of Health, USA).

### RNA sequencing (RNA-seq)

Total RNA of sample was isolated by TRIzol. RNA-seq was performed on the Illumina NovaSeq 6000. Index of the reference genome was built using Hisat2 (v2.0.5) and paired-end reads were aligned to the reference genome using Hisat2 (v2.0.5). The *R* package *“DESeq2”* and *“edgeR”* were used to analyze the data, using a |log2FC| >1 and a *P* value < 0.05 as the cut-off values, respectively.

### Enrichment analysis

Kyoto Encyclopedia of Genes and Genomes (KEGG) pathway and Genome Ontology (GO) analysis was applied to systematically analyze enrichment genes [22–24]. The *R* packages named *clusterProfiler*, *org.Hs.eg.db*, *GOplot*, *stringr* and *tinyarray* were used to implement GO enrichment analysis and KEGG annotations. Using analysis tool (http://www.broadinstitute.org/gsea/index.jsp), Gene Set Enrichment Analysis (GSEA) revealed the overall representation of differential genes in a pathway. A *P* value < 0.05 was considered significantly different.

### Hub genes analysis

Protein and protein interaction (PPI) networks were constructed into Seach Tool for the Retrieval of Interacting Genes (STRING) (https://cn.string-db.org). Then the PPI network was visualized in Cytoscape software. MCC algorithm of the Cyto-Hubba software was used to screen out hub genes.

### Measurement of ATP

The cellular ATP levels were detected by the ATP Assay Kit (Beyotime, China). The cells transfected were lysed by cell lysis buffer and the supernatant was collected after centrifugation at 4℃, 12,000 g. The concentration of the protein was detected by BCA Assay Kit (Beyotime, China) and the assay solution was added and Luminance was measured by multifunctional microplate reader (Tecan Italia Srl, Italy), immediately.

### Serum samples

This study was approved by the Ethics Committee of the Eighth Affiliated Hospital of Sun Yat-sen University and performed according to recommendations of the International Committee of Medical Journal Editors. Written informed consent was obtained from all patients involved. The serum samples of 96 participants were enrolled at the Eighth Affiliated Hospital of Sun Yat-sen University between July 2022 and December 2022, including 35 healthy controls, 34 CHB patients and 27 HBV-HCC patients. Healthy controls were normal liver function, without viral hepatitis infection and other liver-related or malignant disease. The CHB group consisted of patients with chronic HBV infection who have not developed HCC. HBV-HCC group was a histologically confirmed HCC patient diagnosed with CHB (Table S2).

### ELISA

The serum SUV39H1 of participants was measured by the SUV39H1 ELISA kit (LMAI Bio, China). According to the manufacturer’s instructions, the absorption value was read at 450 nm. The SUV39H1 concentration was calculated with reference to standard curves.

### Statistical analysis

SPSS software was used to carry out Spearman’s correlation coefficient. Student’s *t* test was used for two groups’ analyses and one-way ANOVA was for comparing more than two groups to evaluate the statistical significance. In addition, the chi-square test was used to analyze gender differences among three groups. And a receiver operating characteristic (ROC) curve was used to evaluate diagnostic values of SUV39H1 and alpha-fetoprotein (AFP). A *P* value < 0.05 was identified as a significant difference. Analyses were carried out using the GraphPad Prism 8.0.2 software (La Jolla, CA, USA). All cell experiments were repeated three times.

## Results

### SUV39H1 expression is upregulated in HBV-HCC

**Fig. 1:**
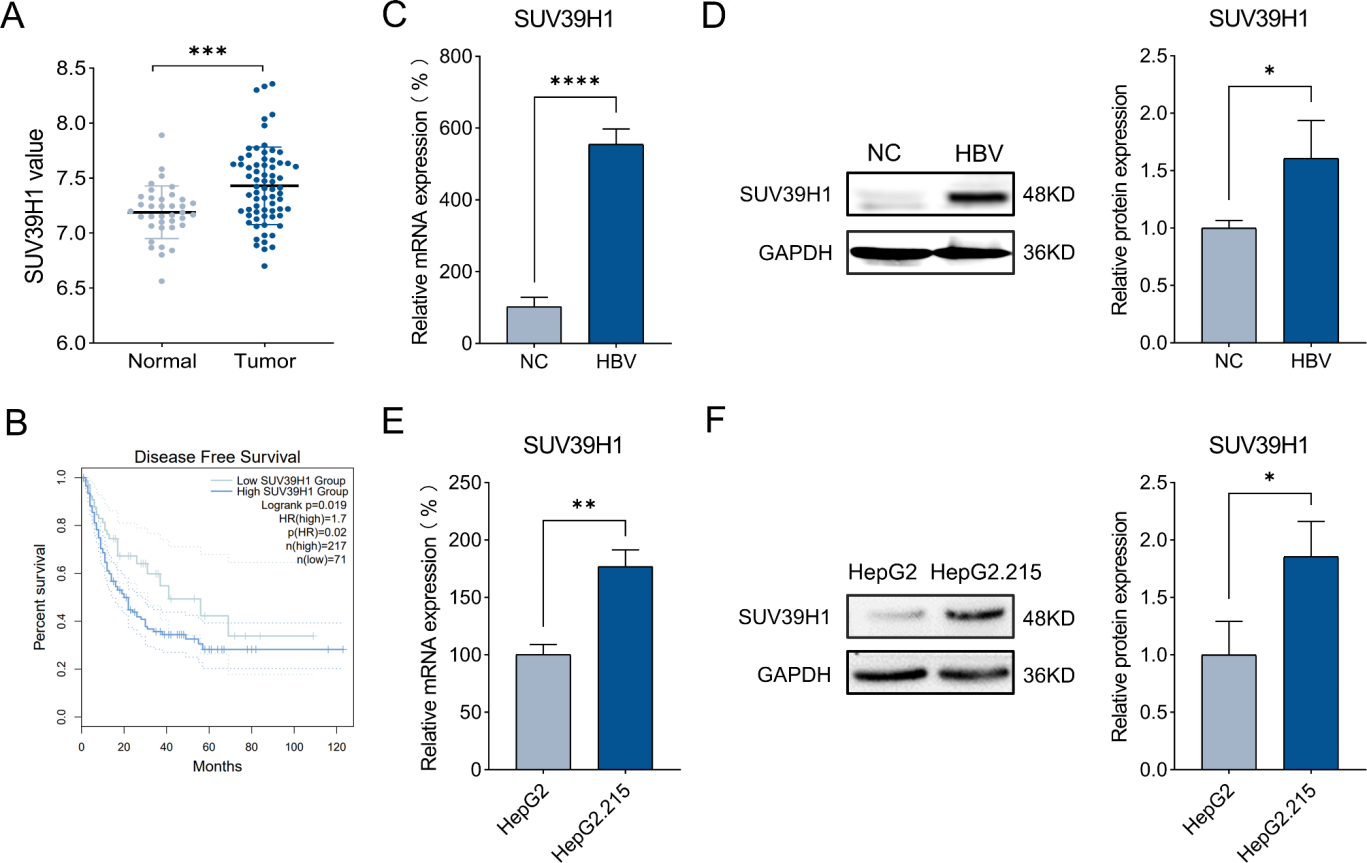
HBV induced SUV39H1 expression in HCC tissues and cells. (**A**) Differential expression of SUV39H1 in HBV-HCC tissues (Tumor) and adjacent normal tissues (Normal). (**B**) The prognostic value of mRNA expression of SUV39H1 in HCC was analyzed by using Kaplan-Meier analysis. (**C, D**) Expression of SUV39H1 mRNA and protein after transfection with HBV plasmid and HBV control plasmid (NC) in HepG2 cells. (**E, F**) Relative level of SUV39H1 mRNA and protein in HepG2 cells and HepG2.215 cells. Data presented as mean ± SEM (n = 3). *P < 0.05, **P < 0.01, ***P < 0.001, ****P < 0.0001 VS Normal, NC or HepG2 cells group

To determine whether HBV infection has a regulatory effect on SUV39H1, the GSE121248 dataset was selected for analysis. The expression of SUV39H1 was significantly increased in HBV-HCC tissues compared with adjacent normal tissues (Fig. 1A). Kaplan-Meier analysis showed that high SUV39H1 expression was correlated with a worse prognosis in HCC patients (Fig. 1B). HBV whole genome plasmid transfection in HepG2 cells significantly increased SUV39H1 mRNA and protein levels compared with the control group (NC) (Fig. 1C-D). Meanwhile, comparing the levels of SUV39H1 in HepG2 cells and HepG2.215 cells also proved that HBV could promote the expression of SUV39H1 (Fig. 1E-F).

### SUV39H1 enhances the proliferation and migration of hepatoma cells

**Fig. 2:**
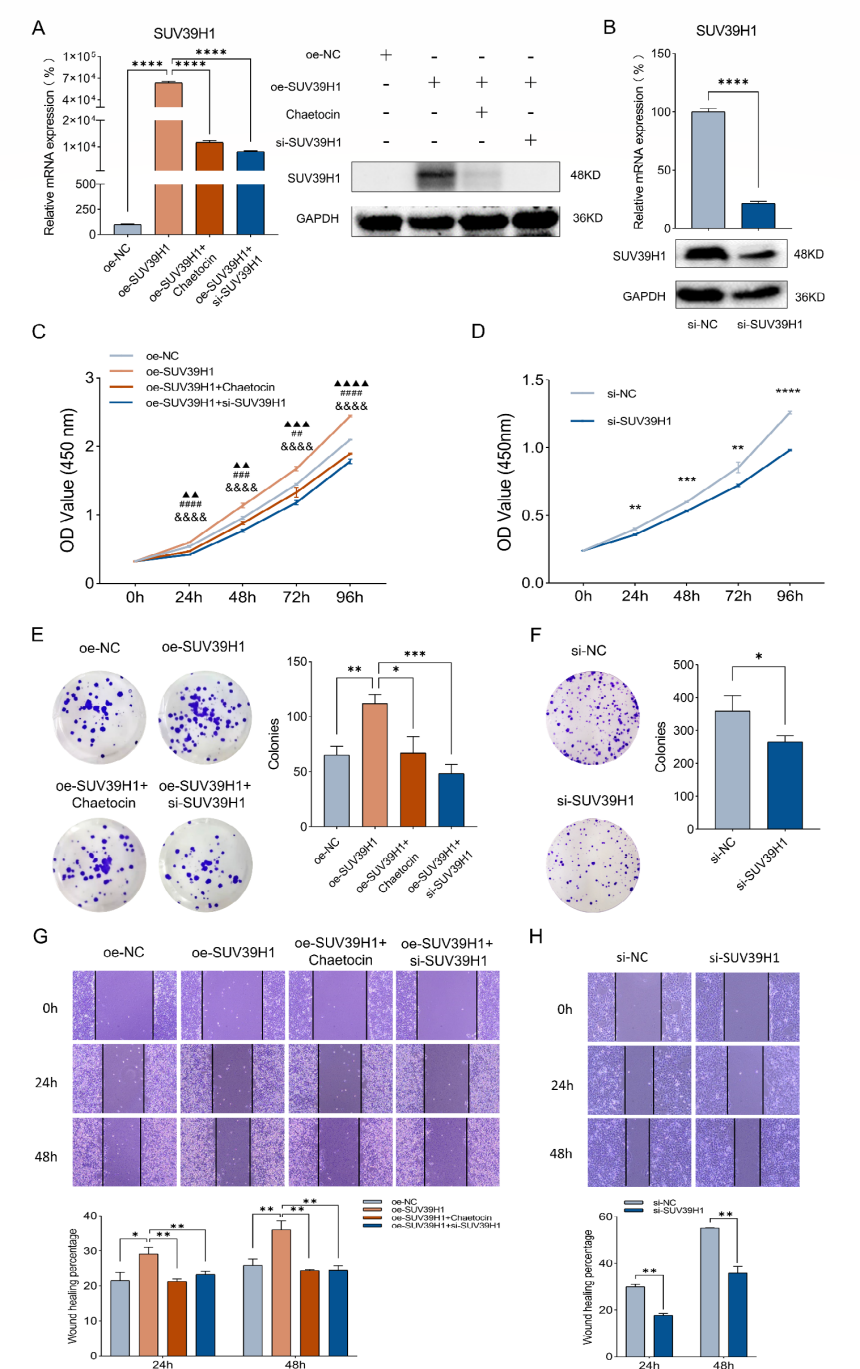
SUV39H1 regulates proliferation and migration of Hep3B cells. (**A**) Expression of SUV39H1 mRNA and protein after transfection with oe-NC, oe-SUV39H1, oe-SUV39H1 + Chaetocin or oe-SUV39H1 + si-SUV39H1 were examined by qPCR and WB. (**B**) The relative expression of SUV39H1 mRNA and protein after transfection with si-NC or si-SUV39H1. (**C–F**) CCK-8 assay and colony growth assay revealed the changes in proliferation ability after above transfection. (**G, H**) Wound healing assay was performed to examine the migration ability. ▲*P* value representing oe-SUV39H1 vs. oe-NC, #oe-SUV39H1 vs. oe-SUV39H1 + Chaetocin, &oe-SUV39H1 vs. oe-SUV39H1 + si-SUV39H1. Data presented as mean ± SEM (n = 3). *P < 0.05, **P < 0.01, ***P < 0.001, ****P < 0.0001, ▲▲*P* < 0.01, ▲▲▲*P* < 0.001, ▲▲▲▲*P* < 0.0001, ##*P* < 0.01, ###*P* < 0.001, ####*P* < 0.0001, &&&&*P* < 0.0001

The function of SUV39H1 in HCC progression was next explored. We established SUV39H1 overexpression and knockdown models in Hep3B cells (Fig. 2A-B). CCK-8 assay and colony growth assay showed that overexpression of SUV39H1 significantly promoted the proliferation of Hep3B cells, while the inhibition of SUV39H1 by Chaetocin or si-SUV39H1 for cells transfected oe-SUV39H1 plasmid reduced the promotion effect (Fig. 2C, E). In contrast, SUV39H1 knockdown markedly inhibited the proliferation of Hep3B cells (Fig. 2D, F). Moreover, wound healing assay revealed that SUV39H1 overexpression significantly promoted the migration of Hep3B cells, while SUV39H1 knockdown inhibited the cell migration (Fig. 2G-H). These results suggest that SUV39H1 is critical for the proliferation and migration of HCC cells.

### OXPHOS is essential pathway regulated by SUV39H1

**Fig. 3:**
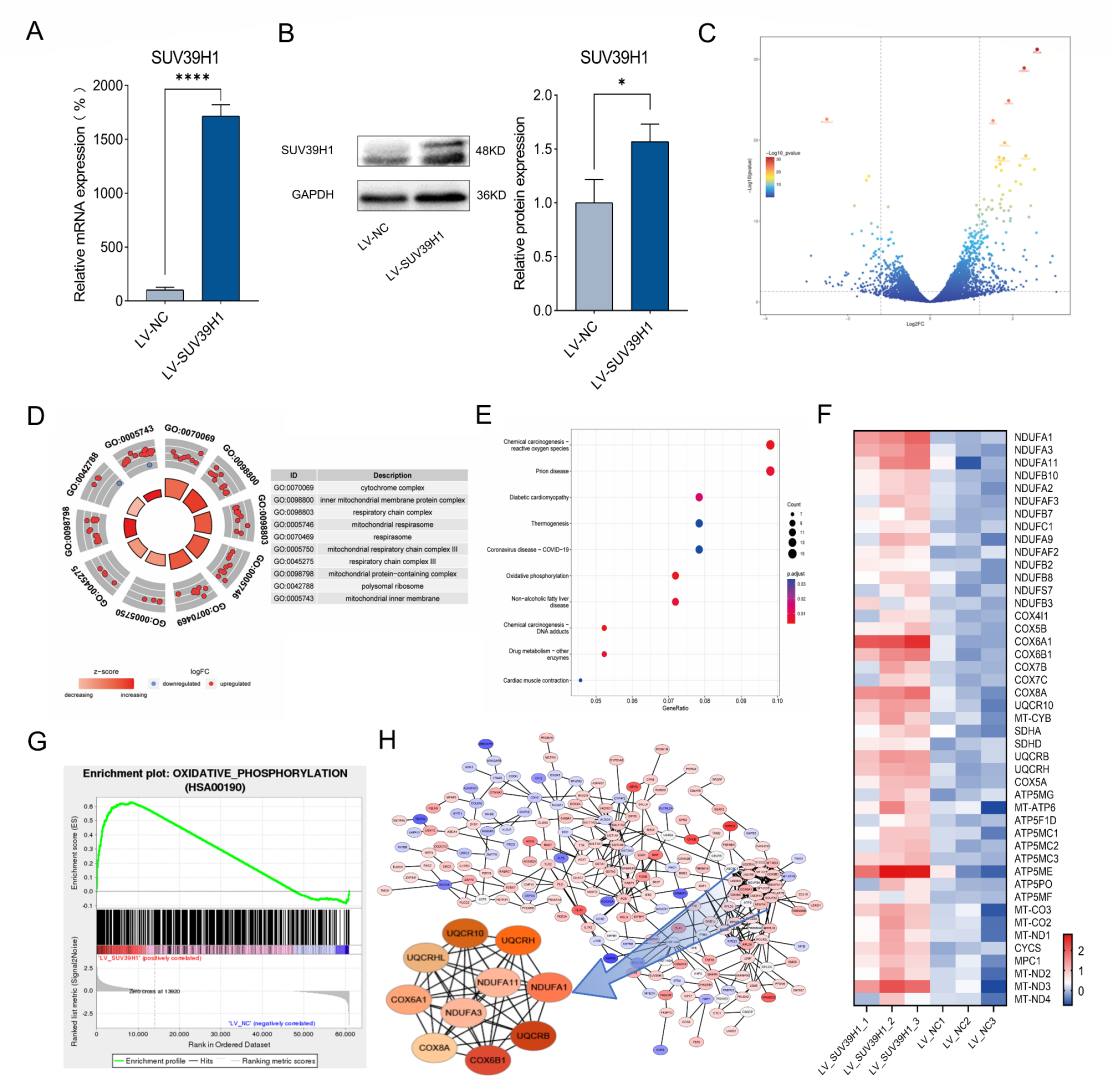
DEGs and enrichment pathway was performed in HepG2 cells stably overexpressing SUV39H1. (**A, B**) Construction of HepG2-LV-SUV39H1, qPCR and WB were used to detect the mRNA and protein of SUV39H1. (**C**) Volcano map showed the differentially expressed genes. Enrichment pathways were analyzed by GO (**D**) and KEGG (**E**) analysis. (**F**) Heatmap revealed that DEGs enriched in the OXPHOS pathway. (**G**) GSEA found a significant difference in OXPHOS pathway. (**H**) Hub genes of PPI network were screened by Cytoscape software

To investigate the molecular mechanism of SUV39H1 promoting proliferation and migration in HCC cells, HepG2 cell stably overexpressing SUV39H1 were established (Fig. 3A-B). The RNA-seq was applied to screen differentially expressed genes (DEGs) in HepG2-LV-SUV39H1 cells versus HepG2-LV-NC cells, including 444 upregulated genes and 218 downregulated genes (Fig. 3C). GO enrichment analysis showed that its function was mainly concentrated in respiratory chain (Fig. 3D). KEGG enrichment analysis also revealed significance in OXPHOS, which including respiratory chain (Fig. 3E). These data showed an important role for OXPHOS in HepG2-LV-SUV39H1 cells. The analysis of OXPHOS pathway genes indicated that 45 genes were upregulated (Fig. 3F). GSEA also confirmed a trend of elevated OXPHOS pathway genes (Fig. 3G). Further, we made PPI network maps of differential genes using Cytoscape software. The top 10 hub genes of DEGs were COX6A1, COX6B1, UQCRB, UQCR10, UQCRH, NDUFA1, NDUFA3, NDUFA11, UQCRHL and COX8A, all associated with genes involved in the mitochondrial respiratory chain (Fig. 3H). These results suggest that SUV39H1 may regulate OXPHOS pathway genes in the mitochondrial respiratory chain.

### SUV39H1 promotes metabolic reprogramming in HCC

**Fig. 4:**
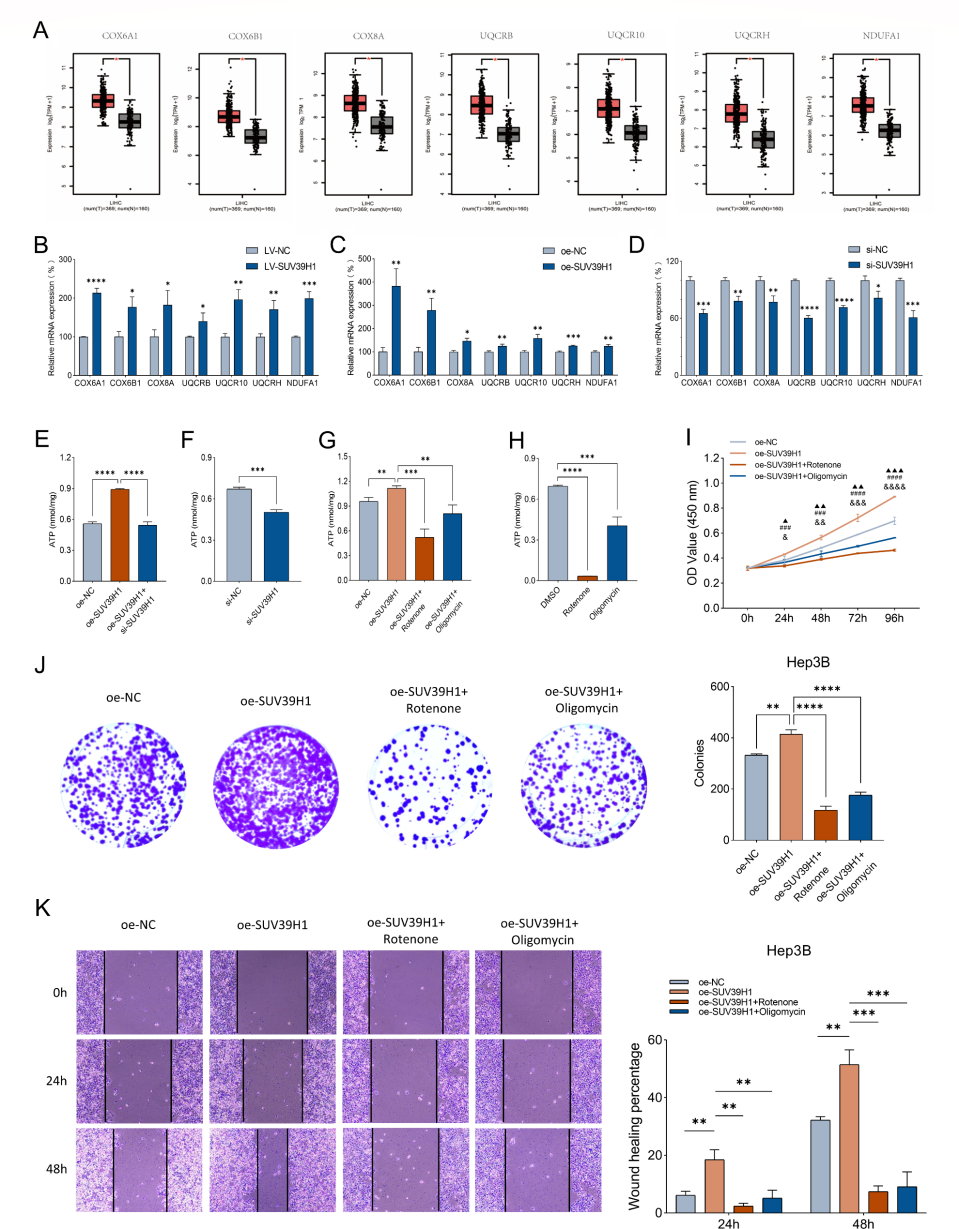
SUV39H1 regulates metabolic reprogramming. (**A**) Difference box plot of hub genes: COX6A1, COX6B1, COX8A, UQCRB, UQCR10, UQCRH, and NDUFA1. Red box means tumors and black box means normal controls. Relative mRNA expression of COX6A1, COX6B1, COX8A, UQCRB, UQCR10, UQCRH, and NDUFA1 in HepG2 cells: (**B**) LV-NC and LV-SUV39H1, (**C**) oe-NC and oe-SUV39H1, (**D**) si-NC and si-SUV39H1. Intracellular ATP detection of HepG2 cells transfected with oe-NC, oe-SUV39H1 or oe-SUV39H1 + si-SUV39H1 for 48 h (**E**), and transfected si-NC or si-SUV39H1(**F**). Intracellular ATP detection of Hep3B cells transfected with oe-NC, oe-SUV39H1, oe-SUV39H1 + Rotenone or oe-SUV39H1 + Oligomycin (**G**), and transfected with DMSO, Rotenone or Oligomycin (**H**). Hep3B cells transfected with oe-NC, oe-SUV39H1, oe-SUV39H1 + Rotenone or oe-SUV39H1 + Oligomycin: (**I**) CCK-8 to detect the proliferation ability; (**J**) Colony growth assay to detect clonal formation; (**K**) Wound healing assay to detect the migration ability. ▲*P* value representing oe-SUV39H1 vs. oe-NC, #oe-SUV39H1 vs. oe-SUV39H1 + Rotenone, &oe-SUV39H1 vs. oe-SUV39H1 + Oligomycin. Data presented as mean ± SEM (n = 3). *P < 0.05, **P < 0.01, ***P < 0.001, ****P < 0.0001, ▲*P* < 0.05, ▲▲*P* < 0.01, ▲▲▲*P* < 0.001, ###*P* < 0.001, ####*P* < 0.0001, &*P* < 0.05, &&*P* < 0.01, &&&*P* < 0.001, &&&&*P* < 0.0001

By searching the expression characteristics of hub genes, we found that COX6A1, COX6B1, COX8A, UQCRB, UQCR10, UQCRH and NDUFA1 were highly expressed in HCC tissues with statistical significance in the TCGA database (Fig. 4A). The mRNA expression of these seven genes in HepG2-LV-SUV39H1 group was proved to be higher than control one (Fig. 4B). Overexpression of SUV39H1 also caused their increase, while silence of SUV39H1 led to their decrease in HepG2 cells (Fig. 4C-D). To investigate the changes of mitochondrial respiratory function, intracellular ATP levels were detected. Compared with control group, ATP levels increased of overexpressing SUV39H1, while co-transfection with si-SUV39H1 restored the increased ATP level in HepG2 cells (Fig. 4E), suggesting that SUV39H1 promotes ATP production in vitro. And ATP level reduced by silencing SUV39H1 in HepG2 cells (Fig. 4F). Both Rotenone and Oligomycin, which were mitochondrial respiratory chain inhibitors, reduced the ATP concentration after overexpression of SUV39H1 in Hep3B cells, and the reduction effect of Rotenone was more obvious (Fig. 4G). In addition, we also examined ATP concentrations in Hep3B cells using Rotenone or Oligomycin alone and found that inhibitors of OXPHOS significantly inhibited ATP production compared to DMSO controls (Fig. 4H).

However, whether SUV39H1 has an effect on hepatoma cells through the OXPHOS pathway remains to be explored. The Hep3B cells transfected with SUV39H1 overexpressed plasmid were treated with Rotenone and Oligomycin, respectively, and CCK-8 and colony growth assays showed that their proliferative capacity was lower than those in the untreated overexpression group (Fig. 4I-J). And the wound healing assay also showed that the ability to migrate was lower than that in the untreated overexpression group (Fig. 4K). It suggested that SUV39H1 might promote metabolic reprogramming by regulating the OXPHOS pathway, thereby improving the proliferation and migration of hepatoma cells.

### Diagnostic value of serum SUV39H1 for HBV-HCC

**Fig. 5:**
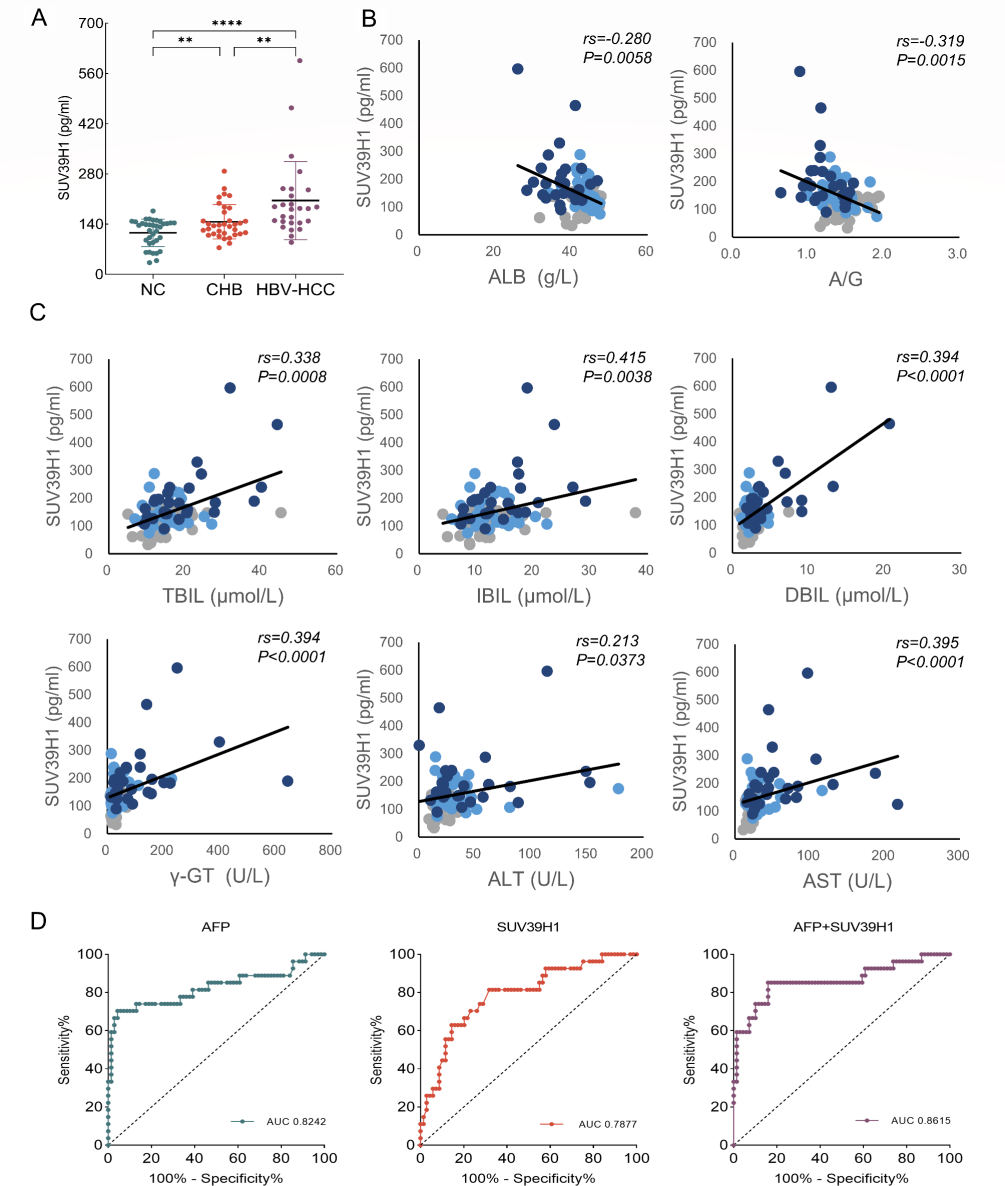
Serum SUV39H1 expression in CHB and HBV-HCC patients and its relationship with clinical characteristics. (**A**) The level of serum SUV39H1 in healthy group (NC), CHB group and HBV-HCC group was determined by ELISA. (**B, C**) Correlation analysis of serum SUV39H1 expression level with biochemical indexes, n = 96. Gray indicates NC group, light blue indicates CHB group and dark blue indicates HBV-HCC group. (**D**) ROC curve showed the distinguishing capability of AFP, SUV39H1 or AFP + SUV39H1 for HBV-HCC patients. **P < 0.01, ****P < 0.0001

To further determine the association between SUV39H1 and HBV-HCC, we examined serum SUV39H1 levels in healthy controls, CHB patients, and HBV-HCC patients. The results showed that the level of SUV39H1 in CHB patients was higher than that in healthy controls, while SUV39H1 expression of HBV-HCC patients was higher than that of CHB patients and healthy controls (Fig. 5A). As shown in Table 1, there were significant decreases in TP, A/G and ALB levels of HBV-HCC group compared with CHB group, while A/G and ALB levels of HBV-HCC group obviously declined contrasted to healthy group. DBIL and γ-GT levels were significantly elevated in HBV-HCC group in contrast to healthy group and CHB group. Comparing with heathy group, ALT and AST levels were increased in CHB group and rose markedly in HBV-HCC group. But only AST displayed a significant increase between CHB group and HBV-HCC group.

**Table 1:** Comparison of characterization between the Healthy group, the CHB group and the HBV-HCC group

Indexes	Healthy group (n = 35)	CHB group(n = 34)	HBV-HCC group (n = 27)	Healthy vs. CHB *P* value	Healthy vs. HBV-HCC *P* value	CHB vs. HBV-HCC *P* value
TP	71.91 ± 0.90	73.92 ± 0.56	68.46 ± 1.31	0.1790	0.1017	**0.0016** **
A/G	1.48 ± 0.04	1.43 ± 0.03	1.22 ± 0.05	0.7007	**0.0000** ****	**0.0013** **
ALB	42.69 ± 0.57	43.35 ± 0.44	37.23 ± 0.96	0.7412	**0.0000** ****	**0.0000** ****
TBIL	15.36 ± 1.17	15.13 ± 0.79	20.07 ± 1.83	0.9977	0.1022	0.0524
DBIL	2.57 ± 0.19	2.61 ± 0.15	5.29 ± 0.84	0.9973	**0.0111** *	**0.0119** *
IBIL	12.79 ± 0.98	12.51 ± 0.66	14.78 ± 1.10	1.0000	0.4123	0.2779
γ-GT	28.78 ± 2.34	34.92 ± 2.84	121.87 ± 26.21	0.7847	**0.0045** **	**0.0095** **
ALT	20.23 ± 1.25	34.02 ± 4.99	45.61 ± 7.63	**0.0323** *	**0.0084** **	0.5070
AST	20.99 ± 0.76	31.07 ± 3.24	60.33 ± 9,74	**0.0133** *	**0.0013** **	**0.0226** *

Values are mean ± SD

TP, total protein; A/G, albumin to globulin; ALB, albumin; TBIL, total bilirubin; DBIL, direct bilirubin; IBIL, indirect bilirubin; γ-GT, γ-glutamyl transpeptidase; ALT, alanine aminotransferase; AST, aspartate transaminase. *P < 0.05, **P < 0.01, ****P < 0.0001

The serum SUV39H1 expression was negatively correlated with ALB, A/G and positively correlated with TBIL, DBIL, IBIL, γ-GT, ALT and AST, indicating that serum SUV39H1 levels had inherent predictive ability for liver injury (Fig. 5B-C). In addition, the SUV39H1 level was positively correlated with serum AFP (Fig. S1).

To validate the diagnostic potential of SUV39H1 and compare it with the commonly used biomarker AFP, we performed ROC curve analysis. As shown in Fig. 5D, the area under the curve (AUC) differentiating HBV-HCC patients from healthy controls and CHB patients was 0.8242 (95%CI: 0.7101–0.9383) for AFP and 0.7877 (95%CI: 0.6848–0.8906) for SUV39H1, indicating that both SUV39H1 and AFP have high diagnostic potential. When SUV39H1 was combined with AFP, the AUC was 0.8615 (95%CI: 0.7644–0.9586), indicating the predictive ability of the two combined diagnoses SUV39H1 and AFP was higher than either one alone. Therefore, SUV39H1 may be an assistant diagnostic marker for AFP.

## Discussion

HBV infection is a major risk factor for HCC, and although large-scale vaccination has greatly reduced HBV infection in children, HBV infection still causes more than 40% of liver cancer [25, 26]. Many mechanisms are involved in this process, including viral DNA integration, viral genome or host gene mutation, epigenetic modification and changes in tumor signaling pathways. Under the synergistic action of these mechanisms, the evolution of HBV infection from inflammation to tumorigenesis is accelerated [27]. Among them, targeted epigenetic modification is considered a promising therapeutic approach. HBV infection can cause epigenetic remodeling through DNA methylation and histone modification, leading to malignant transformation of HCC [28].

SUV39H1 mainly catalyzes H3K9me3 which is generally considered as an epigenetic marker of gene inhibition [6, 7]. Abnormal expression of SUV39H1 has been found in many diseases. For instance, SUV39H1 is highly expressed in renal tubular cells of patients [29]. And the significantly increased expression of SUV39H1 in patients with idiopathic scoliosis can promote the proliferation chondrocytes and Alpinetin can improve colitis by decreasing the expression of SUV39H1 [30, 31]. Previous studies have suggested that SUV39H1 plays a role in tumor inhibition. However, SUV39H1 has also been reported as a tumor promoter in recent years [32]. The inhibition of SUV39H1 in renal clear cell carcinoma can induce iron accumulation and lipid peroxidation, leading to the ferroptosis of cancer cells [33]. It is reported that SUV39H1 enhances the migration ability of HCC cells, and SUV39H1 knockdown impairs the growth and spheroid formation of HCC cells [13]. Besides, SUV39H1 interacts with hepatitis B virus X protein (HBx) to enhance each other’s activity, leading to HBx-mediated hepatocarcinogenesis [14]. However, the carcinogenic mechanism of SUV39H1 in HBV-HCC has not been fully clarified. In this study, we also confirmed that HBV infection could increase the expression of SUV39H1 promoting the proliferation and migration of HBV positive hepatoma cells. We reported that SUV39H1 regulated the pathway genes of the mitochondrial respiratory chain by analyzing the data of LV-oe-SUV39H1 RNA-seq and experimental validation for the first time.

Warburg effect suggests that tumor cells still rely on glycolysis, for energy supply under aerobic conditions, instead of using efficient OXPHOS [34]. However, with the deepening of cancer research, the key role of mitochondrial OXPHOS in tumor progression is being confirmed by more studies [35]. Inhibition of mitochondrial biogenesis in resistant melanoma can induce mitochondrial dysfunction and inhibit tumor bioenergetics, thereby erating intrinsically resistant cells [36]. When the OXPHOS of cancer stem cells (CSCs) was upregulated, the malignant biological behavior of liver cancer stem cells was enhanced [37]. Knockdown of MALAT1 can induce a variety of abnormalities in mitochondrial function, including the reduction of OXPHOS and ATP production, and play a role in promoting HCC [38]. CR6-interacting factor 1 is a mitochondrial protein required for the assembly of OXPHOS complexes and plays an important role in HCC progression [39]. FH535 and Y3 are inhibitors of mitochondrial OXPHOS that disrupt transmembrane potential and electron transport chains, reduce ATP production, and lead to apoptotic hepatoma cells death [40, 41]. However, the role of SUV39H1 in the OXPHOS has not been reported. Through data analysis, we found that the differential genes regulated by SUV39H1 were mainly enriched in OXPHOS pathway.

Since the early diagnosis rate of hepatocellular carcinoma is only 40-50%, and the median survival of advanced hepatocellular carcinoma is only 15 months, the development of early diagnostic markers is the key to improving the prognosis of hepatocellular carcinoma [42]. AFP is currently the most commonly used serum biomarker and the only clinically validated biomarker for HCC, while the sensitivity of AFP for early screening is only 39-64% [43, 44]. Hepatitis B core-related antigen correlates with serum HBV DNA and intrahepatic cccDNA, predicting the occurrence or recurrence of HBV-HCC [45]. Mac-2 binding protein glycosylation isomer (M2BPGi) is a Mac-2 binding protein (M2BP) that is more effective than AFP in forecasting the development of HCC in patients with CHB [46]. Exosomes are intracellularly formed vesicles, encapsulating cell-derived nucleic acid fragments secreted into body fluids such as microRNAs. MicroRNA-21 was significantly higher in the serum of HCC patients than in CHB patients and healthy controls [47]. Serum exosomal heterogeneous nuclear ribonucleoprotein H1 (hnRNPH1) mRNA is a biomarker for HCC, and its diagnostic and predictive power is further increased when coupled with AFP [48]. In addition, blood-free DNA and circulating tumor DNA can also be used as biomarkers to predict the early recurrence of HBV-HCC after HCC resection [49]. Therefore, there is an urgent need for a new highly sensitive serum biomarker for early diagnostic of HBV-HCC. Our data showed that serum SUV39H1 levels in HBV-HCC patients were significantly higher than those in CHB patients and healthy controls. Serum SUV39H1 levels were found to be correlated with liver function indicators and AFP, and the predictive ability of SUV39H1 combined with AFP in the diagnosis of HBV-HCC patients was higher than that of AFP alone. Therefore, serum SUV39H1 may be a diagnostic biomarker for HBV-HCC.

There is no denying the fact that our research also has several limitations. One concern about our findings was the lack of in vivo experiments. Meanwhile, we did not further explore that how SUV39H1 regulates the oxidative phosphorylation pathway. Therefore, future research should be undertaken to explore the regulatory mechanism of SUV39H1 on oxidative phosphorylation pathway through Chromatin Immunoprecipitation and Mass Spectrometry and to make our results more credible by trials in animals.

Our study demonstrated for the first time that elevated SUV39H1 expression could regulate oxidative phosphorylation pathway and the serum level of SUV39H1 indicated the diagnosis of HBV-HCC. These findings provide new sights for the treatment of HBV-HCC. At present, the treatment of HCC is mainly surgery, but there are many newly diagnosed patients with unresectable advanced HCC, resulting in poor survival rate and prognosis. After further exploration, we may be able to use mitochondrial oxidative phosphorylation inhibitors in combination with chemotherapeutic agents to treat advanced HCC patients with high SUV39H1 levels.

## Conclusion

In summary, we confirmed that SUV39H1 is essential in HBV-HCC progression and identified a mechanism by which SUV39H1 targets the OXPHOS pathway. Our data suggest that serum SUV39H1 level may be a biomarker for the diagnosis of HBV-HCC. We are further exploring the role of SUV39H1 in HBV-HCC through animal experiments and investigating other potential targets for SUV39H1 in future studies.

### Electronic supplementary material

Below is the link to the electronic supplementary material.

Supplementary Material 1

Supplementary Material 2

Supplementary Material 3

Supplementary Material 4

## Data Availability

The datasets generated or analyzed in this study are available in open access databases. In this study we used the following databases for analysis, data acquisition and visualization: GEO (https://www.ncbi.nlm.nih.gov/geo/), GEPIA (http://gepia2.cancer-pku.cn/#analysis). For the GEO database, we used the dataset which was coded as GSE121248 (https://www.ncbi.nlm.nih.gov/geo/query/acc.cgi?acc=GSE121248). And our data of RNA-sequencing was coded as GSE237514 (https://www.ncbi.nlm.nih.gov/geo/query/acc.cgi?acc=GSE23751). All data are available from the corresponding author upon reasonable request.

## Abbreviations

SUV39H1: Suppressor of variegation 3–9 homolog 1
HBV-HCC: Hepatitis B virus-associated hepatocellular carcinoma
RNA-seq: RNA sequencing
OXPHOS: Oxidative phosphorylation
CHB: Chronic hepatitis B
AFP: Alpha-fetoprotein
HCC: Hepatocellular carcinoma
HBV: Hepatitis B virus
HCV: Hepatitis C virus
H3K9: Histone 3 lysine 9
ATP: Adenosine triphosphate
ETC: Electron transport chains
DFS: Disease free survival
GEPIA: Gene Expression Profiling Interactive Analysis
siRNA: small interference RNA
HBsAg: hepatitis B surface antigen
DMEM: Dulbecco’s modified Eagle’s medium
FBS: Fetal bovine serum
qPCR: Quantitative real-time PCR
WB: Western blot
HRP: Horseradish peroxidase
CCK-8: Cell Counting Kit-8
KEGG: Kyoto Encyclopedia of Genes and Genomes
PPI: Protein and protein interaction
STRING: Seach Tool for the Retrieval of Interacting Genes
ROC: Receiver operating characteristic
HBsAb: Hepatitis B surface antibody
HBeAg: Hepatitis B e antigen
HBeAb: Hepatitis B e antibody
HBcAb: Hepatitis B core antibody
DEGs: Differentially expressed genes
AUC: Area under the curve
TP: Total protein
A/G: Albumin to globulin
ALB: Albumin
TBIL: Total bilirubin
DBIL: Direct bilirubin
IBIL: Indirect bilirubin
γ-GT: γ-glutamyl transpeptidase
ALT: Alanine aminotransferase
AST: Aspartate transaminase
HBx: Hepatitis B virus X protein
CSCs: Cancer stem cells
M2BPGi: Mac-2 binding protein glycosylation isomer
M2BP: Mac-2 binding protein
hnRNPH1: heterogeneous nuclear ribonucleoprotein H1
