# Supplementary material for: SUV39H1 is a novel biomarker targeting oxidative phosphorylation in hepatitis B virus-associated hepatocellular carcinoma
Source: BMC Cancer. 2023 Nov 28;23:1159. doi: 10.1186/s12885-023-11633-4 (PMC10683103; doi:10.1186/s12885-023-11633-4)
Supplement: Supplementary file 1 — Supplementary Material 1 [file 12885_2023_11633_MOESM1_ESM.pdf]

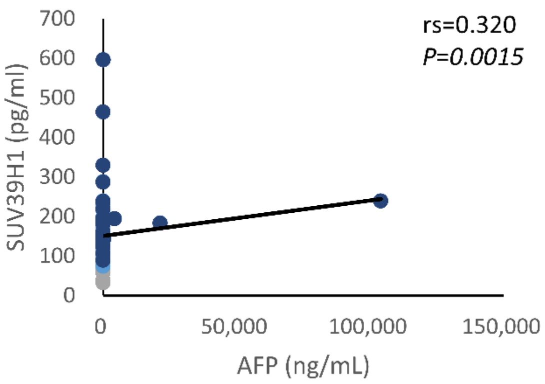


**Supplementary Figure S1.** Spearman's correlation coefficient was performed to analyze the correlation between AFP and SUV39H1, n=96. Related to Figure 5. Gray indicates NC group, light blue indicates CHB group and dark blue indicates HBV-HCC group.
