# Supplementary material for: SUV39H1 is a novel biomarker targeting oxidative phosphorylation in hepatitis B virus-associated hepatocellular carcinoma
Source: BMC Cancer. 2023 Nov 28;23:1159. doi: 10.1186/s12885-023-11633-4 (PMC10683103; doi:10.1186/s12885-023-11633-4)
Supplement: Supplementary file 2 — Supplementary Material 2 [file 12885_2023_11633_MOESM2_ESM.pdf]

Supplementary Table S1 Sequences of si-RNA and primers

Name	Sequences
si-NC sense	5′-UUCUCCGAACGUGUCACGUT-3′
si-NC antisense	5′-ACGUGACACGUUCGGAGAAT -3′
si-SUV39H1 sense	5′-CCUUCGUGUACAUCAAUGATT-3′
si-SUV39H1 antisense	5′-UCAUUGAUGUACACGAAGGTT-3′
GAPDH Forward primer[19]	5′-GGACCTGACCTGCCGTCTAG-3′
GAPDH Reverse primer[19]	5′-GTAGCCCAGGATGCCCTTGA-3′
SUV39H1 Forward primer	5′-TCCGCACCCTGGAGAAGATT-3′
SUV39H1 Reverse primer	5′-ACGTCCTCCACGTAGTCCAG-3′
COX6A1 Forward primer	5′-GTAGTTGGTGTGTCCTCGGTTTCTC-3′
COX6A1 Reverse primer	5′-TCTCCGTGGTGCGACTTCAGG-3′
COX6B1 Forward primer	5′-CCCCAACCAGAACCAGACTAGAAAC-3′
COX6B1 Reverse primer	5′-CCTCCTTTAGCGGTCATTGCCTTC-3′
COX8A Forward primer	5′-GTTGGGCTTACCTCCTGCTTCG-3′
COX8A Reverse primer	5′-CACTCTGGCCTCCTGTAGGTCTC-3′
UQCRB Forward primer	5′-ATGTGAATTCATGGCTGGTTGGTAAGCAGGCC-3′
UQCRB Reverse primer	5′-ATGCCTCGAGCTTCTTTGCCCATTCTTC-3′
UQCR10 Forward primer	5′-TCCACCTTCGCCCTCACCATC-3′
UQCR10 Reverse primer	5′-CCTCGTTGATGTGGTCGTAGATAGC-3′
UQCRH Forward primer	5′-CAATGCGAGCAGTTGGAGAAATGTG-3′
UQCRH Reverse primer	5′-CCTCCGTGCAATCCTCTTCTGTATG-3′
NDUFA1 Forward primer	5′-CTACTGCGTACATCCACAGGTTCAC-3′
NDUFA1 Reverse primer	5′-GATCAACTCCAGAGATGCGCCTATC-3′
