# Supplementary material for: SUV39H1 is a novel biomarker targeting oxidative phosphorylation in hepatitis B virus-associated hepatocellular carcinoma
Source: BMC Cancer. 2023 Nov 28;23:1159. doi: 10.1186/s12885-023-11633-4 (PMC10683103; doi:10.1186/s12885-023-11633-4)
Supplement: Supplementary file 3 — Supplementary Material 3 [file 12885_2023_11633_MOESM3_ESM.pdf]

Supplementary Table S2 Characterization of HBV infection between the Healthy group, the CHB group and the HBV-HCC group

Indexes		Healthy group（n=35）	CHB group（n=34）	HBV-HCC group（n=27）	Healthy vs CHB *P* value	Healthy vs HBV-HCC *P* value	CHB vs HBV-HCC *P* value
Age		52.69±2.04	52.71±1.49	57.19±2.56	1.0000	0.4385	0.3601
Gender	Male	24 (68.57%)	25 (73.53%)	22 (81.48%)	0.7918	0.3807	0.5491
	Female	11 (31.43%)	9 (26.47%)	5 (18.52%)			
HBsAg		-	863.39±187.10	866.20±195.51（n=21）	-	-	-
HBsAb		-	29.59±9.55（n=8）	19.60±15.97（n=3）	-	-	0.9465
HBeAg		-	78.61±48.15（n=34）	13.89±9.71（n=20）	-	-	0.4804
HBeAb		-	0.52±0.16（n=34）	0.20±0.11（n=20）	-	-	0.2682
HBcAb		-	0.01±0.00（n=34）	0.02±0.01（n=20）	-	-	0.6991

Values are mean ±SD.

HBsAg, hepatitis B surface antigen; *HBsAb*, hepatitis B surface antibody; *HBeAg*, hepatitis B e antigen; *HBeAb*, hepatitis B e antibody; *HBcAb*, hepatitis B core antibody.
