# Supplementary material for: SUV39H1 is a novel biomarker targeting oxidative phosphorylation in hepatitis B virus-associated hepatocellular carcinoma
Source: BMC Cancer. 2023 Nov 28;23:1159. doi: 10.1186/s12885-023-11633-4 (PMC10683103; doi:10.1186/s12885-023-11633-4)
Supplement: Supplementary file 4 — Supplementary Material 4 [file 12885_2023_11633_MOESM4_ESM.pdf]

SUPPLEMENTARY FIGURE S2 Expression of SUV39H1 protein after transfection with HBV plasmid and HBV control plasmid (NC) in HepG2 cells. Related to Figure 1D.

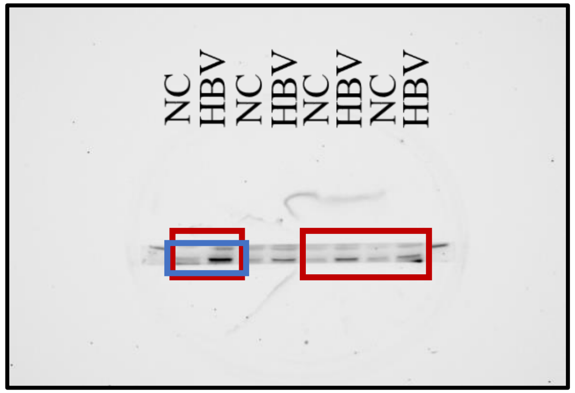

SUV39H1（48KD）

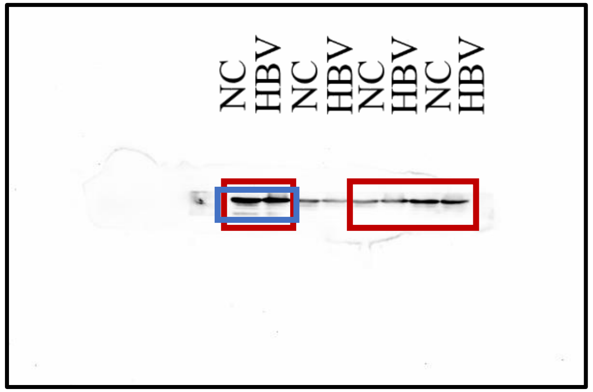

GAPDH（36KD）

（Notice： The red marked bands were used for statistical analysis and blue for Figure display. ）

SUPPLEMENTARY FIGURE S3 Expression of SUV39H1 protein in HepG2 cells and HepG2.215 cells. Related to Figure 1F.

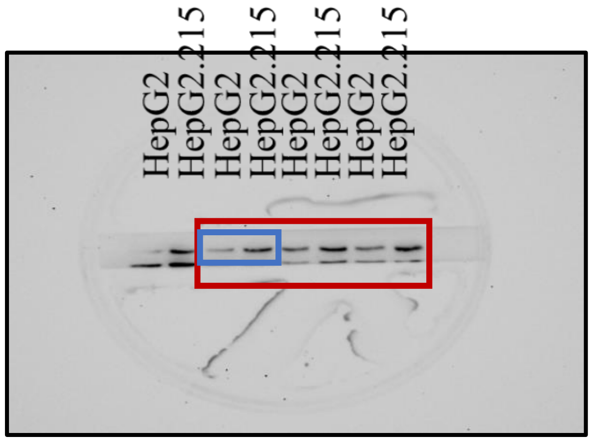

SUV39H1（48KD）

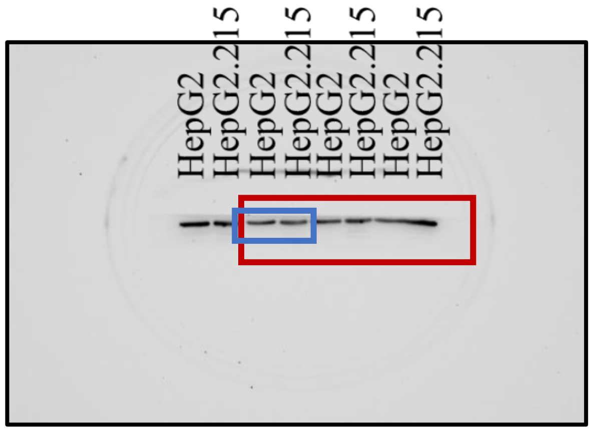

GAPDH（36KD）

（Notice：The red marked bands were used for statistical analysis and blue for Figure display. ）

SUPPLEMENTARY FIGURE S4 Expression of SUV39H1 protein after transfection with oe-NC, oe-SUV39H1, oe-SUV39H1+Chaetocin or oe-SUV39H1+si-SUV39H1 were examined WB. Related to Figure 2A.

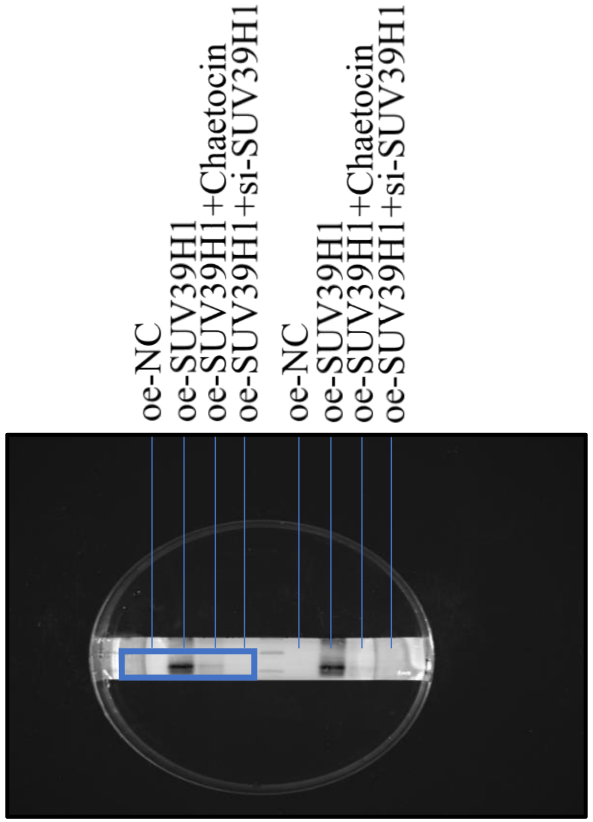

SUV39H1 （48KD）

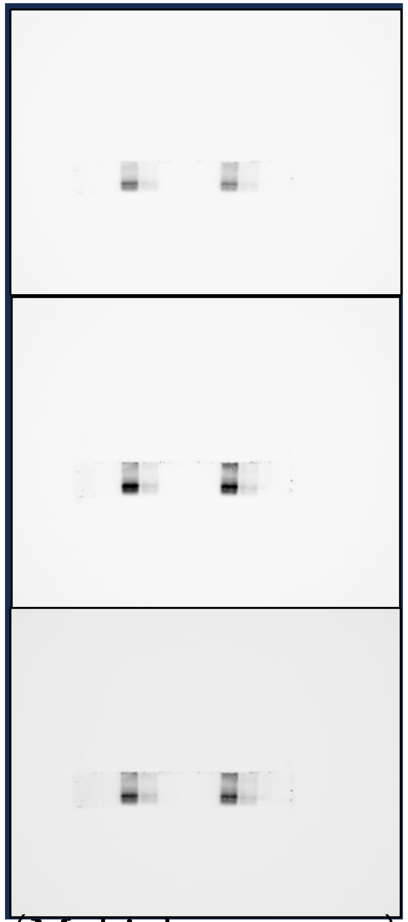

（Multiple exposure）

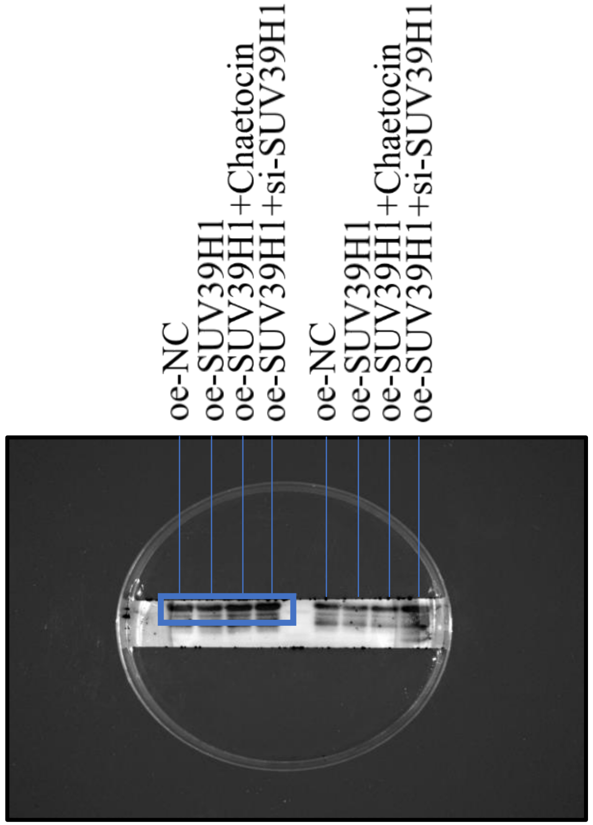

GAPDH （36KD）

（Notice： The blue marked bands were used for Figure display. ）

SUPPLEMENTARY FIGURE S5 The relative expression of SUV39H1 protein after transfection with si-NC or si-SUV39H1. Related to Figure 2B.

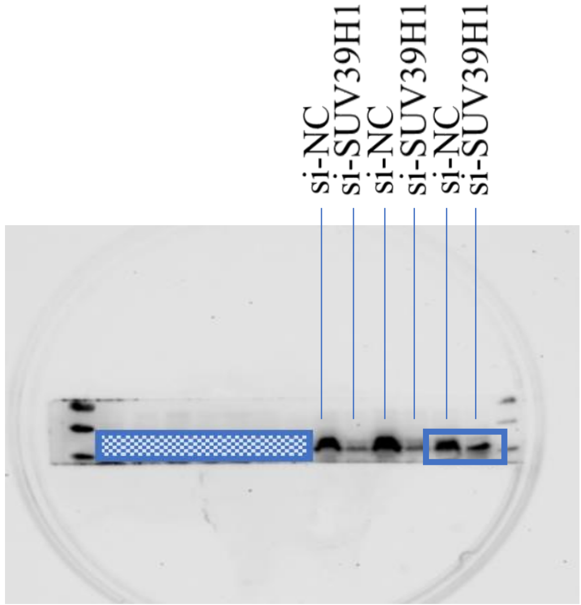

SUV39H1（48KD）

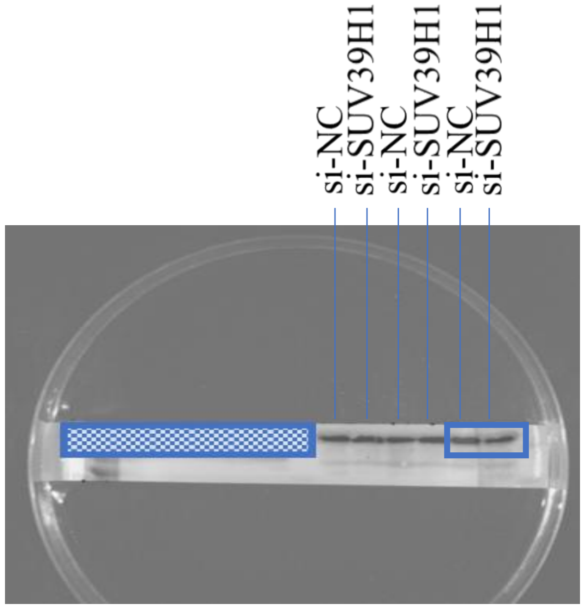

GAPDH（36KD）

（Notice：The blue marked bands were used for Figure display.）

SUPPLEMENTARY FIGURE S6 Construction of HepG2-LV-SUV39H1, WB was used to detect the protein of SUV39H1. Related to Figure 3B.

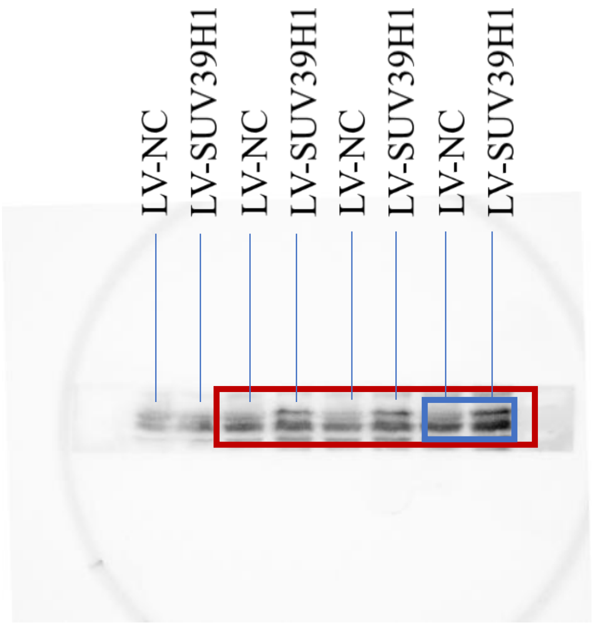

SUV39H1（48KD）

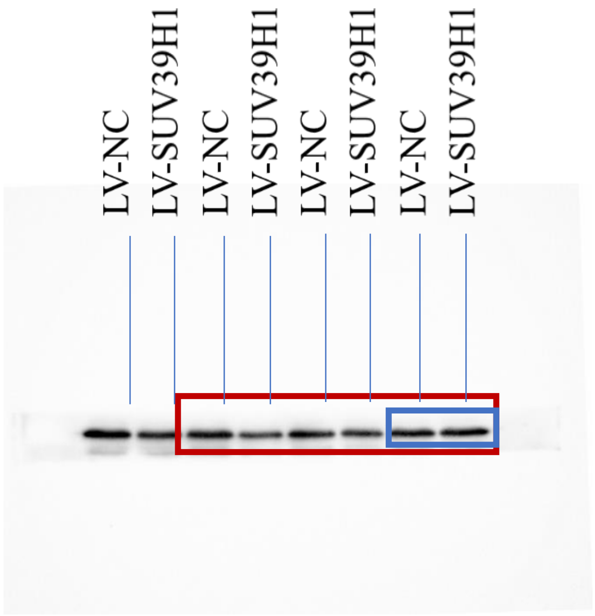

GAPDH（36KD）

（Notice：The red marked bands were used for statistical analysis and blue for Figure display.）
